# Excellent Interrater Reliability for Manual Segmentation of the Medial Perirhinal Cortex

**DOI:** 10.3390/brainsci13060850

**Published:** 2023-05-24

**Authors:** Nicolas A. Henzen, Julia Reinhardt, Maria Blatow, Reto W. Kressig, Sabine Krumm

**Affiliations:** 1 University Department of Geriatric Medicine FELIX PLATTER, 4055 Basel, Switzerland; 2 Faculty of Psychology, University of Basel, 4001 Basel, Switzerland; 3 Division of Diagnostic and Interventional Neuroradiology, Department of Radiology, University Hospital Basel, University of Basel, 4031 Basel, Switzerland; 4 Department of Cardiology and Cardiovascular Research Institute Basel (CRIB), University Hospital Basel, University of Basel, 4031 Basel, Switzerland; 5 Department of Orthopedic Surgery and Traumatology, University Hospital of Basel, University of Basel, 4031 Basel, Switzerland; 6 Section of Neuroradiology, Department of Radiology and Nuclear Medicine, Neurocenter, Cantonal Hospital Lucerne, University of Lucerne, 6000 Lucerne, Switzerland; 7 Faculty of Medicine, University of Basel, 4056 Basel, Switzerland

**Keywords:** Alzheimer’s disease, perirhinal cortex, entorhinal cortex, segmentation, structural MRI

## Abstract

*Objective:* Evaluation of interrater reliability for manual segmentation of brain structures that are affected first by neurofibrillary tau pathology in Alzheimer’s disease. *Method:* Medial perirhinal cortex, lateral perirhinal cortex, and entorhinal cortex were manually segmented by two raters on structural magnetic resonance images of 44 adults (20 men; mean age = 69.2 ± 10.4 years). Intraclass correlation coefficients (ICC) of cortical thickness and volumes were calculated. *Results:* Very high ICC values of manual segmentation for the cortical thickness of all regions (0.953–0.986) and consistently lower ICC values for volume estimates of the medial and lateral perirhinal cortex (0.705–0.874). *Conclusions:* The applied manual segmentation protocol allows different raters to achieve remarkably similar cortical thickness estimates for regions of the parahippocampal gyrus. In addition, the results suggest a preference for cortical thickness over volume as a reliable measure of atrophy, especially for regions affected by collateral sulcus variability (i.e., medial and lateral perirhinal cortex). The results provide a basis for future automated segmentation and collection of normative data.

## 1. Introduction

Detecting the earliest signs of Alzheimer’s disease (AD) is a great challenge since the progressive neurodegenerative disease usually remains unnoticed until difficulties in daily living activities emerge [1]. At this stage, neuropathological brain changes in domains such as memory, language, executive processing, or visuospatial functioning are objectified with standard neuropsychological assessments [2,3]. However, neuropathological brain changes associated with AD are thought to begin years before the onset of clinical symptoms. The main neuropathological markers of AD are β-amyloid plaques and neurofibrillary tangles (NFT) of pathological tau protein [4]. In comparison to the extracellular amyloid plaques, intracellular NFT occur more numerously and in a more hierarchical pattern in the temporal lobe [5]. NFT disturb neuronal functioning and are thought to be more strongly correlated to cognitive impairment than amyloid plaques [5,6,7]. The continuous deposition of NFT in AD is closely associated with neuron loss [4,8] and is believed to be causally linked to cerebral atrophy [9]. NFT density is indirectly reflected by grey matter atrophy in structural magnetic resonance imaging (MRI) [10]. Against this background, the present study focuses on NFT. In typical AD, this neurofibrillary tau pathology starts in the medial part of the perirhinal cortex (mPRC), also referred to as the transentorhinal cortex [4,11]. As the disease progresses, NFT spread to the entorhinal cortex (ERC) and eventually to hippocampal subfields and throughout the brain [4,12]. In support of this notion, Sone et al. [13] found regional NFT accumulation in early-stage AD to be associated with cortical thinning in the perirhinal cortex and ERC. Hippocampal atrophy, on the other hand, was more strongly associated with a higher number of β-amyloid plaques [13]. Accordingly, sensitive preclinical structural imaging biomarkers enable the earliest diagnosis and, thus, treatment with current symptomatic as well as future disease-modifying drugs when as little damage as possible has been done [2,14]. Furthermore, the earliest neuropathological predictors allow the tracking of disease progression and are of significant interest in AD research as well as the clinical setting [15].

Regions of the parahippocampal gyrus (e.g., ERC, mPRC) of the medial temporal lobe are associated with atrophy in the early stages of AD. As mentioned before, in typical AD, the neurofibrillary tau pathology starts in the mPRC before spreading to the ERC and hippocampus [4,11]. Nonetheless, in clinical settings, AD diagnosis commonly relies on atrophy scores of the ERC [16] as well as on medial temporal lobe atrophy scores rating the hippocampus, the choroid fissure, and the lateral ventricle [17]. The mPRC is mostly neglected. Since the mPRC is a fairly small structure (e.g., length of 2.1 cm anterior-posterior and mean width of 0.95 cm in one random cognitively healthy participant segmented by NAH), visual, quantitative evaluation is difficult. However, the assessment of the mPRC integrity in clinical settings could be improved by a computed atrophy value (e.g., cortical thickness). Recent studies support the potential of the mPRC’s integrity in clinical settings. For example, Kulason et al. [18] detected significant differences in mPRC but not ERC thickness between individuals with mild cognitive impairment (MCI) and normal controls. Further results revealed cortical thinning in the mPRC 9–14 years prior to an MCI diagnosis, while ERC thinning was measurable 8–11 years prior to diagnosis [19]. In comparison, hippocampus atrophy was found 2–4 years before the clinical symptom onset of AD [20]. Moreover, a study published by Krumm et al. [21] reported patients with amnestic MCI, the presumed prodromal stage of Alzheimer’s dementia, to show atrophy of the mPRC before the lateral part of the perirhinal cortex (lPRC). Specifically, the lPRC was only atrophied in patients with Alzheimer’s dementia. These findings are in line with the proposed staging of NFT accumulation [4,12] and suggest mPRC integrity as a promising marker to detect the earliest signs of AD. Furthermore, the evaluation of mPRC integrity allows a differentiation to other neurodegenerative diseases in which NFT neuropathology plays a key role but does not primarily affect the mPRC, such as corticobasal degeneration with prominent NFT in the cerebral cortex, specifically in frontoparietal regions [22]. Interpretation of the literature on the perirhinal cortex is harmed by the use of different nomenclature and several changes in the definition of neuroanatomic localization [23]. Braak and Braak first described a transitional area between the ERC and neocortex, namely the transentorhinal region. This region is characterized by a distinct layer of oblique pyramidal cells in layer II, which are highly vulnerable to neurofibrillary changes and are the first to be affected in typical AD [4,24]. According to Taylor and Probst [11], the mPRC coincides with the transentorhinal area by Braak and Braak [24]. Further, the anterior and posterior borders of the mPRC correspond to Brodmann’s cytoarchitectonic field 35 [25]. The highly variable nomenclature and differences in defining the areas  boundaries make comparison between studies difficult.

As mentioned above, cortical NFT begin in the mPRC, while the lPRC is only affected in a later stage [4]. This reflects the importance of separately segmenting these structures. Otherwise, disease progression may be underestimated in early-stage AD. Anatomically, the collateral sulcus of the medial temporal lobe defines the transition from mPRC to lPRC and is characterized by high inter- and intraindividual differences (e.g., length and form of the sulcus). This collateral sulcus variability represents a significant obstacle to the accurate segmentation of the mPRC and lPRC, as it substantially influences the delimitation of the borders of these regions [11,26]. Specifically, the lack of software that accurately and automatically segments the mPRC might be the main reason why imaging studies have hardly ever investigated the mPRC separately from the lPRC. A reliable manual segmentation protocol as the basis for future development and validation of an automated segmentation protocol is of significant importance [27].

Comparable to the focus of structural assessment in AD, neuropsychological testing routinely focuses on hippocampal function (e.g., episodic memory tests). As the site of the first NFT, perirhinal functioning might reflect a promising neuropsychological marker in incipient AD. However, specific tasks for perirhinal cortex measurement are not yet readily available. The majority of our current knowledge about the perirhinal cortex results from animal studies. These indicate that the perirhinal cortex is located at the apex of the ventral stream and is associated with visual object processing [28,29,30]. Originating from the primary visual cortex, the complexity of represented features increases along the ventral visual stream leading to the perirhinal cortex, which is in charge of the most complex visual analyses [28,31,32]. In addition, the perirhinal cortex is connected to various other brain regions and further receives and processes auditory and somatosensory information [33]. A promising oddity detection task testing the ability to form an integrated perceptual representation from similar low-level visual features, a function assigned to the perirhinal cortex, was recently published by Frei et al. [34]. The task reflects a sensitive measure to differentiate early-stage AD patients from cognitively healthy adults. Further, based on the conceptual structure account by Taylor et al. [35], the perirhinal cortex is crucial in the discrimination of highly confusable (e.g., living things) in comparison to less confusable (e.g., non-living things) stimuli. Accordingly, AD patients are expected to show more difficulty processing living than non-living things. This is based on the fact that living things share more similar features, making them easier to confuse than non-living things. Such function of semantic object processing can be assessed by fluency tests. A study by Hirni et al. [36] showed that semantic fluency tests can monitor mPRC integrity. Another study by Krumm et al. [37] demonstrated that the combined measurement of two fluency tests of living things (animals and fruits) best differentiated AD patients and cognitively healthy participants. Overall, these results are very promising and reflect the relevance of further research addressing the perirhinal cortex/mPRC on the challenging path to detect the earliest signs of AD.

In summary, the present study aimed to evaluate the interrater reliability of a manual segmentation protocol, which takes collateral sulcus variability into account (depicted in [21]). Regions of interest (ROIs) for the manual segmentation comprised mPRC, lPRC, and ERC, which are affected in different stages of classic AD and might allow an evaluation of disease progression [4]. High interrater reliability would indicate that the applied manual segmentation protocol allows different raters to achieve highly similar results. In addition, the manual segmentation was carried out by an experienced (SK) and an inexperienced (NAH) rater to assess the practicability of the applied segmentation protocol. Furthermore, we contrasted the interrater reliability of cortical thickness and volume estimates. In comparison to three-dimensional measurements of atrophy (i.e., volumes), cortical thickness of the perirhinal cortex is independent of the form and length of the collateral sulcus [38,39,40]. Therefore, we expect differences in manual segmentation between the two raters to be more pronounced for volume estimates.

The main object of this study was to: (1) evaluate the interrater reliability of a manual segmentation protocol for the mPRC, lPRC, and ERC between two raters with different levels of experience and (2) compare two measurements of atrophy estimates (cortical thickness vs. volume).

## 2. Materials and Methods

### 2.1. Participants

In a clinical setting, a potential future automated segmentation protocol would be applied to patients with different diseases. To satisfy this circumstance, data from 44 native Swiss-German or German-speaking adults (20 men; mean age = 69.2 years, SD = 10.42 years) were randomly selected from a larger study (N = 131) at the Memory Clinic FELIX PLATTER, University Department of Geriatric Medicine FELIX PLATTER, Basel, Switzerland. The random selection process was performed by NAH while fully blinded to participant information (i.e., diagnosis, age, gender, and education). Only after manual segmentation was NAH unblinded, and participants were assigned to 1 of 4 groups, namely cognitively healthy normal controls (NCs), Major Depression (MD), MCI or dementia due to AD, and MCI or dementia due to other etiologies than AD (non-AD; e.g., due to Lewy body disease). See Table 1 for a more detailed overview. Cognitively healthy NCs were recruited from the “Registry of Healthy Individuals Interested to Participate in Research” of the Memory Clinic University Department of Geriatric Medicine FELIX PLATTER Basel, Switzerland. Thorough medical screening ensured their neurologic and psychiatric health (exclusion criteria: severe sensory or motor deficits; severe visual, auditory or speech deficits; severe systemic disease; diseases with severe or probable impact on the central nervous system [e.g., neurologic disorders including cerebral-vascular diseases, generalized atherosclerosis, and psychiatric problems]; continuous mild-to intense pain; and intake of potent psychoactive substances, except minor tranquilizers). In addition, they were not allowed to have more than 1 score out of the normal range on the Mini-Mental State Examination (MMSE; [41]), the Basel Verbal Learning Test (BVLT, the German equivalent to the California Verbal Learning Test; [42]), Trail Making Test B [43], or the 16-items version of the Informant Questionnaire on Cognitive Decline in the Elderly [44]. All tests were administered in Swiss German or German. The AD group consisted of nine patients diagnosed with Alzheimer’s dementia according to DSM-IV [45] and NINCDS-ADRDA criteria [46] and nine patients diagnosed with amnestic MCI [47] according to DSM-IV [45] and Winblad et al. [48] criteria. The non-AD group contained three patients diagnosed with dementia due to other etiologies than AD (two due to Lewy body disease and one due to Parkinson’s disease) and six patients diagnosed with non-amnestic MCI (five with unknown etiology and one due to vascular disease). AD and non-AD patients were recruited from the Memory Clinic FELIX PLATTER, University Department of Geriatric Medicine FELIX PLATTER, Basel, Switzerland, where they received a neuropsychological assessment, MRI, medical and neurological examinations including blood analyses, and gait analysis.

Some patients additionally received cerebrospinal fluid testing and/or positron emission tomography scans. AD and non-AD patients were diagnosed by an interdisciplinary team at the University Department of Geriatric Medicine FELIX PLATTER, Basel, Switzerland [49]. Six patients were diagnosed with MD by the same interdisciplinary team mentioned above, and 2 were recruited from the University Psychiatric Clinics Basel, Switzerland. MDs were diagnosed according to ICD-10 criteria [50] and evaluated in an interview and with standardized questionnaires. MD patients had to score at least 6 points on the Geriatric Depression Scale [51], 10 or more points on the Becks Depression Inventory [52], or at least 13 points on the Becks Depression Inventory-II [53]. The study was approved by the local ethics committee and performed in compliance with relevant laws and institutional guidelines. Written informed consent was obtained from all participants prior to participation.

### 2.2. MRI Acquisition

All participants received T1-weighted 3D magnetization-prepared rapid acquisition gradient echo (MPRAGE) structural MRI using the same 3-Tesla scanner (MAGNETOM Skyra fit, Siemens (Erlangen, Germany); 12 channel headcoil; inversion time = 900 ms, repetition time 2300 ms, echo time 2.92 ms, flip angle = 9°; acquisition matrix = 256 × 256 mm, voxel size = 1 mm isotropic) at the University Hospital Basel, Switzerland.

### 2.3. Preprocessing of Structural MR Images

MRI scans were preprocessed using FreeSurfer (Massachusetts General Hospital, Boston, MA, USA; http://surfer.nmr.mgh.harvard.edu (accessed on 8 January 2022); [38,39]). In a semi-automated processing stream, FreeSurfer segmented the T1-weighted 3D magnetization-prepared rapid acquisition gradient echo (MPRAGE) volumes into the grey and white matter. Then, the surface of white matter, represented by the transition area from white to grey matter, and the pial surface were modeled [38]. Finally, tissue classification was visually verified, and, if necessary, manual correction was carried out for all subjects.

### 2.4. Manual Segmentation

**Figure 1:**
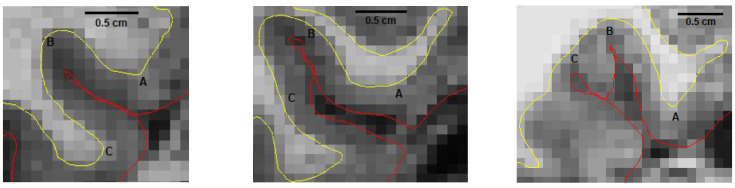
Example of three different appearances of the collateral sulcus on the right hemisphere in random participants and their effects on borders of medial and lateral perirhinal cortex; left: normal collateral sulcus length (<1.5 cm), middle: deep collateral sulcus length (>1.5 cm), and right: bifurcated collateral sulcus. A. medial border of mPRC, B. lateral border of mPRC/medial border of lPRC, C. lateral border of lPRC. The yellow line represents the grey matter’s surface, and the red line represents the pial surface as generated by FreeSurfer. Abbrevations: mPRC = medial perirhinal cortex; lPRC = lateral perirhinal cortex.

**Figure 2:**
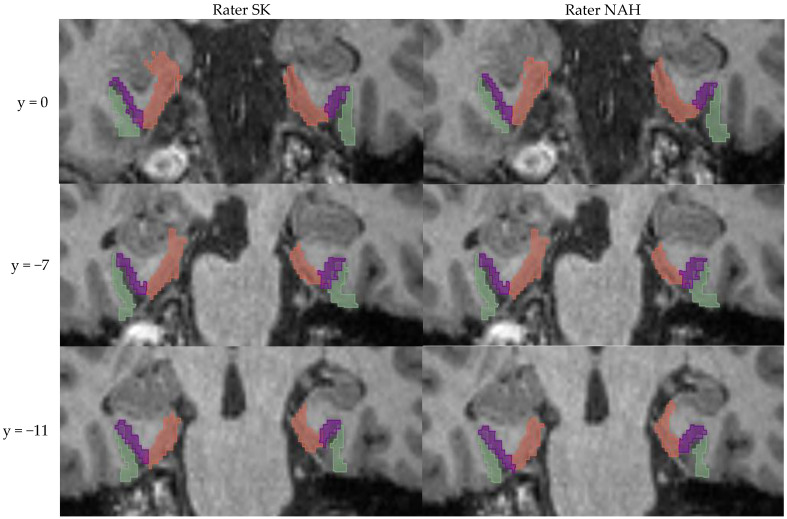
Examples of manual segmentation (using FreeSurfer) for three different coronal slices (MNI y-coordinates are shown) of the same random participant. Green = lateral perirhinal cortex; lila = medial perirhinal cortex; orange = entorhinal cortex.

ROIs (i.e., mPRC, lPRC, and ERC) for both hemispheres were manually drawn by 2 raters blinded for diagnosis, including 1 experienced rater (SK) and 1 inexperienced rater (NAH). Introduction to the manual segmentation for the inexperienced rater included the demonstration of landmarks for all ROIs in FreeSurfer by SK according to the protocol depicted in Krumm et al. [21] (for example, the anterior-posterior borders of manual segmentation, see Supplementary Materials). Afterward, manual segmentation was performed autonomously using the manual segmentation protocol on coronal slices of native-space reconstructions of the cortical surface provided by FreeSurfer (see Figure 1 and Figure 2 for examples). Mean cortical thickness and volumes for each ROI were acquired using FreeSurfer (Massachusetts General Hospital, Boston, MA, USA; http://surfer.nmr.mgh.harvard.edu).

### 2.5. Statistical Analyses

To evaluate the interrater reliability between both raters, ROI cortical thickness and volume estimates from all participants were compared between the 2 raters. Thus, we did not differentiate between diagnostic groups. Intraclass correlation coefficient (ICC) estimates and their 95% confidence intervals were calculated using SPSS 22 (IBM Corp. Released 2013. IBM SPSS Statistics for Windows, Version 22.0. Armonk, NY, USA) based on a single-rating, absolute-agreement, and a 2-way mixed-effects model according to the guidelines of Koo and Li [54].

## 3. Results

The results of the ICC analyses for cortical thickness estimates are summarized in Table 2. Analogous results for volume estimates are depicted in Table 3.

## 4. Discussion

We aimed to evaluate the interrater reliability of a manual segmentation protocol for regions of the medial temporal lobe, including the mPRC, lPRC, and ERC. Especially the mPRC is of great interest because this brain structure is the first region affected by neurofibrillary tau pathology in typical AD [4]. The ICC analyses for cortical thickness estimates showed very high interrater reliability for manual mPRC segmentation as well as for lPRC and ERC. The results provide evidence that the applied segmentation protocol, which considers collateral sulcus variability, allows different raters to achieve strikingly similar results. Furthermore, after only a brief introduction to the landmarks of all ROIs, the inexperienced rater (NAH) was able to follow the segmentation protocol as depicted in Krumm et al. [21], reflecting remarkable practicability.

As mentioned before, the collateral sulcus of the medial temporal lobe defines the boundaries between the mPRC and lPRC and is characterized by high anatomical variability (e.g., length and form of the sulcus). Therefore, the difficulty in segmentation is most likely associated with the variable shape of the collateral sulci [11,26]. In addition, the collateral sulcus variability mostly affects the volume and much less the cortical thickness of adjacent areas [38,39,40]. For the mPRC and lPRC, the ICC analysis for volume compared to cortical thickness estimates revealed a consistently lower degree of reliability between both raters. This difference was not found for the ERC, whose borders are not as strongly determined by the collateral sulcus. These results are in line with previous findings describing the effect of the collateral sulcus variability on volumes of its adjacent regions [40,55]. In summary, the lower ICC results for volume estimates suggest cortical thickness to be a more reliable and, thus, preferable measurement of atrophy, particularly for regions affected by the collateral sulcus variability (e.g., mPRC and lPRC).

Nonetheless, there are limitations to our study. We did not aim to reflect the general population but rather to illustrate the clinical routine, where most of the individuals are patients with different etiologies, and only a few are healthy. Participants were randomly selected from a larger study. This led to a heterogeneous sample with a sufficient total number of investigated subjects but rather low sample sizes per diagnostic group (e.g., NCs, MD). Furthermore, the random selection of participants resulted in a sample of predominantly well-educated individuals who were rather young compared to the usual age of individuals suffering from dementia. However, as can be seen from the mean MMSE scores in Table 1, they were in very early disease stages. Future studies could increase the sizes of diagnostic groups to differentially investigate them. In addition, a sample that represents the general population in terms of age, ethnicity, multimorbidity, and educational background should be aimed for. Further, we renounced investigating the intrarater agreement because we do not expect significant intrarater differences since our interrater reliability is very high. However, future studies might still want to include outcomes of more than two raters and analyze the intrarater reliability to improve the generalizability of the results. Further, we used isotropic 1 mm^3^ T1-weighted images for our analysis. Since the cortical thickness is automatically generated after manual segmentation, tissue misclassification during the preprocessing steps (e.g., the border between white matter and grey matter) is possible. This could lead to inaccurate values of cortical thickness, which may hinder research and application in the clinical setting. Recent findings suggest the use of higher-resolution images for hippocampus subfields, which may also apply to our parahippocampal regions and could improve results [27].

Manual segmentation is time-consuming and unfeasible for clinical settings, let alone for larger data. Software that accurately and automatically segments mPRC would be of great interest. Xie et al. [56] proposed a promising, automated segmentation based on a multiatlas segmentation software for Brodmann area 35, which corresponds to the mPRC. The comparison between the manual and automated segmentation showed a Dice similarity coefficient of 0.77 [56]. This result reflects room for improvement for automated segmentation of the mPRC, which could possibly be achieved using the manual segmentation protocol applied in our study (depicted in [21]) as ground truth. As mentioned before, a reliable manual segmentation protocol is of utmost importance for the development and validation of an automated segmentation protocol [27]. In combination with commonly used atrophy scores in clinical settings (e.g., ERC and medial temporal lobe atrophy [16,17]), mPRC integrity has the potential to add valuable information, especially in early-stage AD, for follow-up examinations as well as for differential diagnostics. To achieve this, a proof of concept needs to be stated that the mPRC is atrophied in AD individuals only but intact in patients suffering from other diseases. Since the collateral sulcus has a pronounced effect on borders of adjacent regions (e.g., mPRC) and shows very different appearances not only between subjects but also within the same individual, the next step is the collection of normative data from healthy individuals as well as different diagnostic groups. This allows not only to capture the interindividual variability of healthy individuals but also to determine pathological values of atrophy (e.g., cortical thickness) for individualized patient evaluation in clinical routine. This would suggest mPRC’s integrity to be used as an early biomarker for AD and provide additional information for differential diagnostics in clinical settings. In addition, as the site of the first NFT, a broader focus on the assessment of perirhinal cortex function for the early diagnosis of AD in clinical settings would be worthwhile. The combined evaluation of structural and functional changes in clinical settings and research represents a promising approach to detecting the earliest signs of AD. Furthermore, future functional anatomical studies may provide more insight into these early changes in AD.

## 5. Conclusions

This study provides evidence that the applied manual segmentation protocol for cortical regions first affected by neurofibrillary tau pathology in AD (e.g., the mPRC and ERC) allows different raters to achieve remarkably similar cortical thickness estimates. In addition, we confirmed the practicability for an inexperienced rater and finally provided evidence to prefer cortical thickness to volume as a reliable measure of atrophy, especially for regions affected by the collateral sulcus variability (e.g., the mPRC and lPRC). Future studies are encouraged to develop time-saving automated segmentation on the basis of the provided results and compare the accuracy with the manual segmentation. Further, the present study recommends a manual segmentation protocol to be used in future proof of concept studies. Such studies should prove the hypothesis that the mPRC is affected early by atrophy in AD but not in the early stages of other neurodegenerative diseases. It is essential to apply the segmentation to a broad range of diseases using appropriate sample sizes to allow the generalization of results. Moreover, for individualized patient evaluation in clinical routine, the collection of normative data from healthy individuals as well as different diagnostic groups is needed.

## Figures and Tables

**Table 1:** Sample characteristics.

	NCs (n = 9)		AD (n = 18)		non-AD (n = 9)		MD (n = 8)	
Gender (m/f)	5/4		7/11		6/3		2/6	
	Mean	SD	Mean	SD	Mean	SD	Mean	SD
Age (years)	70.89	11.41	71.56	10.61	71.56	7.09	59.13	6.40
Education (years)	13.33	4.03	14.50	3.24	12.89	2.42	13.63	2.88
MMSE score	29.00	1.00	26.72	2.42	25.89	1.54	29.00	1.41

Note. NCs = normal controls; AD = Alzheimer’s disease; non-AD = cognitive impairment due to other etiologies than Alzheimer’s disease; MD = Major Depression; m = male; f = female; MMSE = Mini-Mental State Examination.

**Table 2:** ICC calculation for cortical thickness estimates using single-rating, absolute-agreement, two-way mixed-effects model.

Variable	Cronbach’s Alpha	ICC	95% Confidence Interval		F Test		
			Lower Bound	Upper Bound	Value	df1/df2	*p*
mPRC lh	0.993	0.986	0.974	0.992	136.618	43/43	4.81 × 10^−35^
mPRC rh	0.994	0.985	0.967	0.992	154.670	43/43	3.46 × 10^−36^
lPRC lh	0.992	0.984	0.970	0.991	118.834	43/43	9.23 × 10^−34^
lPRC rh	0.975	0.953	0.915	0.974	40.344	43/43	5.82 × 10^−24^
ERC lh	0.984	0.969	0.944	0.983	62.657	43/43	6.44 × 10^−28^
ERC rh	0.980	0.961	0.930	0.979	49.390	43/43	9.02 × 10^−26^

Note. ICC = intraclass correlation coefficient; mPRC = medial perirhinal cortex; lPRC = lateral perirhinal cortex; ERC = entorhinal cortex; lh = left hemisphere; rh = right hemisphere.

**Table 3:** ICC calculation for volume estimates using single-rating, absolute-agreement, two-way mixed-effects model.

Variable	Cronbach’s Alpha	ICC	95% Confidence Interval		F Test		
			Lower Bound	Upper Bound	Value	df1/df2	*p*
mPRC lh	0.932	0.874	0.781	0.929	14.647	43/43	3.06 × 10^−15^
mPRC rh	0.864	0.757	0.597	0.859	7.363	43/43	6.58 × 10^−10^
lPRC lh	0.909	0.831	0.712	0.904	10.960	43/43	6.53 × 10^−13^
lPRC rh	0.825	0.705	0.518	0.827	5.703	43/43	3.96 × 10^−8^
ERC lh	0.978	0.948	0.887	0.974	44.603	43/43	7.40 × 10^−25^
ERC rh	0.951	0.908	0.838	0.949	20.326	43/43	5.58 × 10^−18^

Note. ICC = intraclass correlation coefficient; mPRC = medial perirhinal cortex; lPRC = lateral perirhinal cortex; ERC = entorhinal cortex; lh = left hemisphere; rh = right hemisphere.

## Data Availability

All analysis code and research materials, as well as metadata and/or full data, are available upon request.

## Supplementary Materials

The following supporting information can be downloaded at: https://www.mdpi.com/article/10.3390/brainsci13060850/s1, Figures S1–S8: Visualization of the borders of mPRC, lPRC, and ERC in coronal slices.
