# Supplementary material for: Excellent Interrater Reliability for Manual Segmentation of the Medial Perirhinal Cortex
Source: Brain Sci. 2023 May 24;13(6):850. doi: 10.3390/brainsci13060850 (PMC10296215; doi:10.3390/brainsci13060850)
Supplement: Supplementary file 1 [file brainsci-13-00850-s001.zip › brainsci-2361298-S-materials.pdf]

brain sciences

MDPI

Supplementary materials

The following **Figures S1-8** visualize the borders of mPRC, lPRC, and ERC in coronal slices using FreeSurfer in one random participant. **A**: How to find the **anterior border** of the left hemisphere (**Figures S1-4)**. Yellow line represents white matter surface and red line represents pial surface as generated by FreeSurfer. *Note: 1slice is equal to 1mm.*

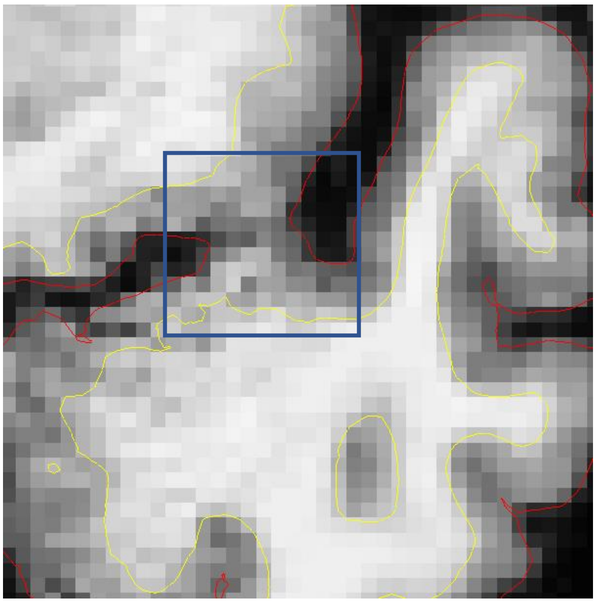

Figure S1. Anterior border +3slices: Last slice, where the white matter of the limen insulae is not visible (blue mark).

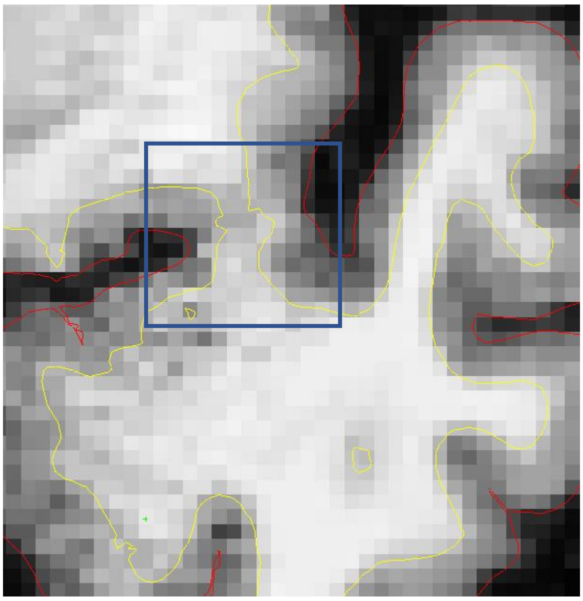

Figure S2. Anterior border +2slices: First slice, where the white matter of the limen insulae is visible (blue mark).

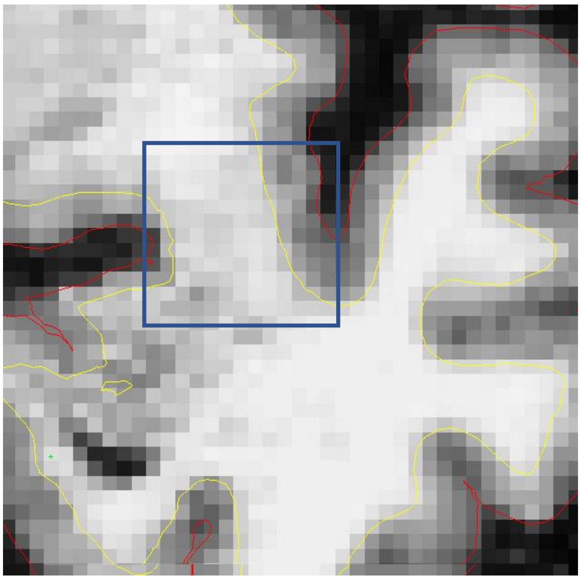

Figure S3. Anterior border: 2slices posterior to the first slice, where the white matter of the limen insulae is visible.

Structure	Anterior border
ERC	2 mm posterior to the first anterior slice where the white matter of the limen insulae is visible.
Medial PRC	Same coronal level as the anterior border of the ERC, i.e., 2 mm posterior to the first anterior slice where the white matter of the limen insulae is visible.
Lateral PRC	Same coronal level as the anterior border of the ERC, i.e., 2 mm posterior to the first anterior slice where the white matter of the limen insulae is visible.

Figure S4. Anterior border: Adapted and modified from Krumm et al. (2016).

B: How to find the **posterior border** of the left hemisphere (**Figures S5-8**).

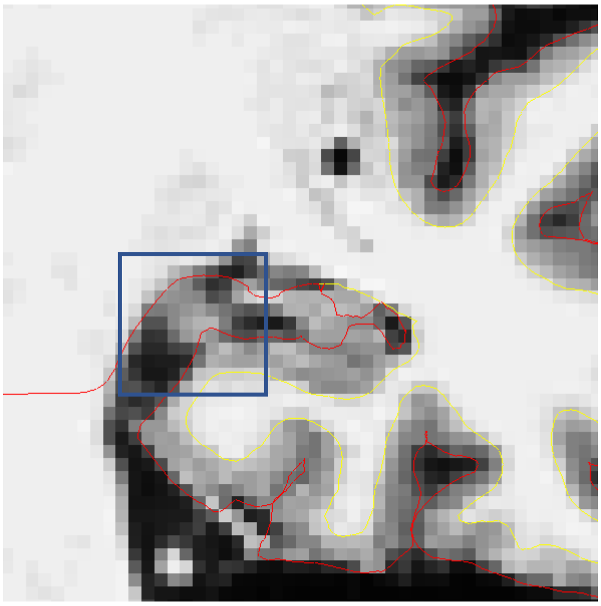

Figure S5. Posterior border +2slices: Apex of the intralimbic gyrus is still visible (blue mark).

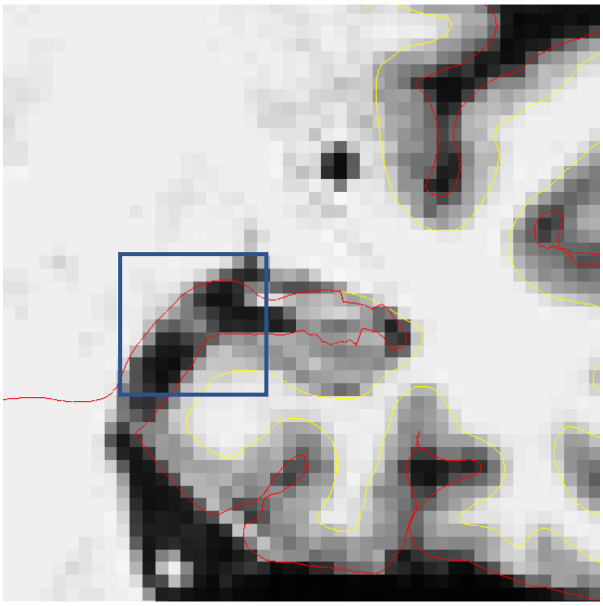

Figure S6. Posterior border +1slice: Last slice still containing the apex of the intralimbic gyrus (blue mark).

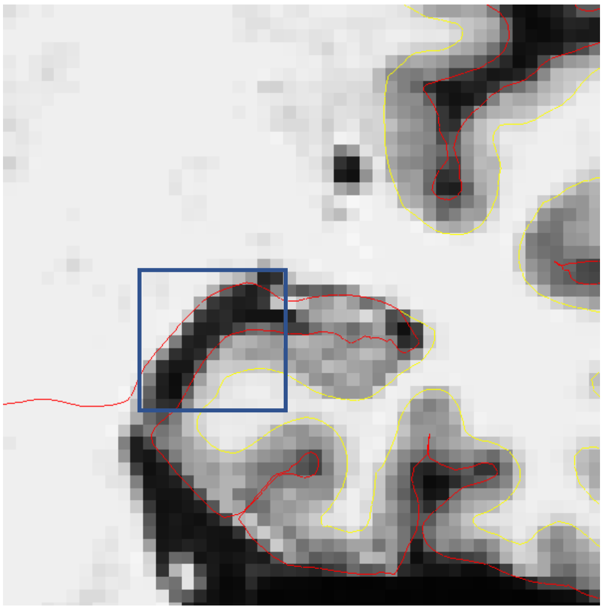

Figure S7. Posterior border: 1slice posterior to the last slice still containing the apex of the intralimbic gyrus (blue mark).

Structure	Posterior border
ERC	1 mm posterior to the last slice still containing the apex of the intralimbic gyrus.
Medial PRC	1 mm posterior to the last slice still containing the apex of the intralimbic gyrus.
Lateral PRC	1 mm posterior to the last slice still containing the apex of the intralimbic gyrus.

Figure S8. Posterior border: Adapted and modified from Krumm et al. (2016).
